# Large language models in patient education: a scoping review of applications in medicine

**DOI:** 10.3389/fmed.2024.1477898

**Published:** 2024-10-29

**Authors:** Serhat Aydin, Mert Karabacak, Victoria Vlachos, Konstantinos Margetis

**Affiliations:** ^1^School of Medicine, Koç University, Istanbul, Türkiye; ^2^Department of Neurosurgery, Mount Sinai Health System, New York, NY, United States; ^3^College of Human Ecology, Cornell University, Ithaca, NY, United States

**Keywords:** large language models, ChatGPT, patient education, artificial intelligence, machine learning, deep learning

## Abstract

**Introduction:**

Large Language Models (LLMs) are sophisticated algorithms that analyze and generate vast amounts of textual data, mimicking human communication. Notable LLMs include GPT-4o by Open AI, Claude 3.5 Sonnet by Anthropic, and Gemini by Google. This scoping review aims to synthesize the current applications and potential uses of LLMs in patient education and engagement.

**Materials and methods:**

Following the PRISMA-ScR checklist and methodologies by Arksey, O’Malley, and Levac, we conducted a scoping review. We searched PubMed in June 2024, using keywords and MeSH terms related to LLMs and patient education. Two authors conducted the initial screening, and discrepancies were resolved by consensus. We employed thematic analysis to address our primary research question.

**Results:**

The review identified 201 studies, predominantly from the United States (58.2%). Six themes emerged: generating patient education materials, interpreting medical information, providing lifestyle recommendations, supporting customized medication use, offering perioperative care instructions, and optimizing doctor-patient interaction. LLMs were found to provide accurate responses to patient queries, enhance existing educational materials, and translate medical information into patient-friendly language. However, challenges such as readability, accuracy, and potential biases were noted.

**Discussion:**

LLMs demonstrate significant potential in patient education and engagement by creating accessible educational materials, interpreting complex medical information, and enhancing communication between patients and healthcare providers. Nonetheless, issues related to the accuracy and readability of LLM-generated content, as well as ethical concerns, require further research and development. Future studies should focus on improving LLMs and ensuring content reliability while addressing ethical considerations.

## 1 Introduction

Large Language Models (LLMs) are sophisticated algorithms that analyze and generate extensive textual data (1). These models leverage vast corpora of unlabeled text and incorporate reinforcement learning from human feedback to discern syntactical patterns and contextual nuances within languages. Consequently, LLMs can produce responses that closely mimic human communication when presented with diverse, open-ended queries (2–4). Several notable LLMs have emerged recently, including GPT-4o by Open AI (5), Claude 3.5 Sonnet by Anthropic (6), and Gemini by Google (7).

LLMs have demonstrated significant potential in medicine, with transformative applications across various domains, including clinical settings. These AI-powered systems can streamline clinical workflows, help with clinical decision-making, and ultimately improve patient outcomes. Recent studies highlight the utility of LLMs in clinical decision support, providing valuable insights that enable healthcare teams to make more informed treatment decisions (8–10). LLMs also show promise as educational tools by enhancing the quality and accessibility of materials. However, from a patient’s perspective, they present both opportunities and risks. The varying levels of medical knowledge among patients may impede their ability to critically assess the information provided by LLMs, unlike clinicians who are trained to do so.

As of July 2024, there was limited synthesis of knowledge regarding the evidence base, applications, and evaluation methods of LLMs in patient education and engagement. This scoping review aims to address this gap by mapping the available literature on potential applications of LLMs in patient education and identifying future research directions. Our primary research question is: “What are the current and potential uses of LLMs in patient education and engagement as described in the literature?” This review seeks to enhance future discussions on using LLMs for patient care, including education, engagement, workload reduction, patient-centered health customization, and communication.

## 2 Materials and methods

This study employed a scoping review methodology, adhering to the Preferred Reporting Items for Systematic Reviews and Meta-Analyses Extension for Scoping Reviews (PRISMA-ScR) checklist (11). The review process was based on the methodological framework developed by Arksey and O’Malley (12), with further refinements as proposed by Levac et al. (13).

### 2.1 Literature search

A literature search was conducted in June 2024 using the PubMed database. The search strategy, detailed in Supplementary Methods S1, combined relevant keywords and Medical Subject Headings (MeSH) terms related to LLMs and patient education.

### 2.2 Study selection

Citation management was facilitated by Covidence software (Veritas Health Innovation). The inclusion criteria encompassed studies addressing the use, accuracy, relevance, or effectiveness of LLMs in patient education, patient engagement, answering patient-specific questions, or generating patient education materials. Studies were excluded if they did not primarily focus on LLMs for patient education, engagement, or answering patient questions; did not assess LLMs in healthcare settings or had only indirect relations to patients; or focused solely on technical aspects or architecture of LLMs without considering their application in patient education or engagement. A detailed description of the inclusion and exclusion criteria is provided in Supplementary Methods S2.

The selection process involved two stages. In the initial screening, two authors (SA and VV) independently reviewed the titles and abstracts of retrieved articles. Studies passing the initial screening were then read in full by both authors. Studies deemed eligible by both reviewers were included in the analysis. In cases of disagreement, a third author (MK) was consulted to resolve discrepancies.

### 2.3 Thematic analysis

We employed thematic analysis, following the methodology proposed by Braun and Clarke (14), to address our primary research question. The process began with an author (SA) reading and coding 25 randomly selected articles, focusing on content related to the potential uses of LLMs in patient education and engagement. Subsequently, two authors (SA and MK) examined the remaining manuscripts, seeking additional themes or data that could either reinforce or challenge the established themes. This iterative process facilitated further refinement of the themes through group discussions centered on patient education and engagement.

## 3 Results

### 3.1 Literature search

The initial search strategy yielded 661 papers. After removing one duplicate, 660 papers remained for screening. Based on title and abstract screening, 365 papers (55.3%) were excluded. Full-text review was conducted for 295 papers (44.7% of the initial pool), resulting in 201 papers (30% of the initial pool) meeting the study inclusion criteria (Supplementary Figure S1). Supplementary Data S1 presents all of the included papers.

### 3.2 Descriptive analysis

**Figure 1:**
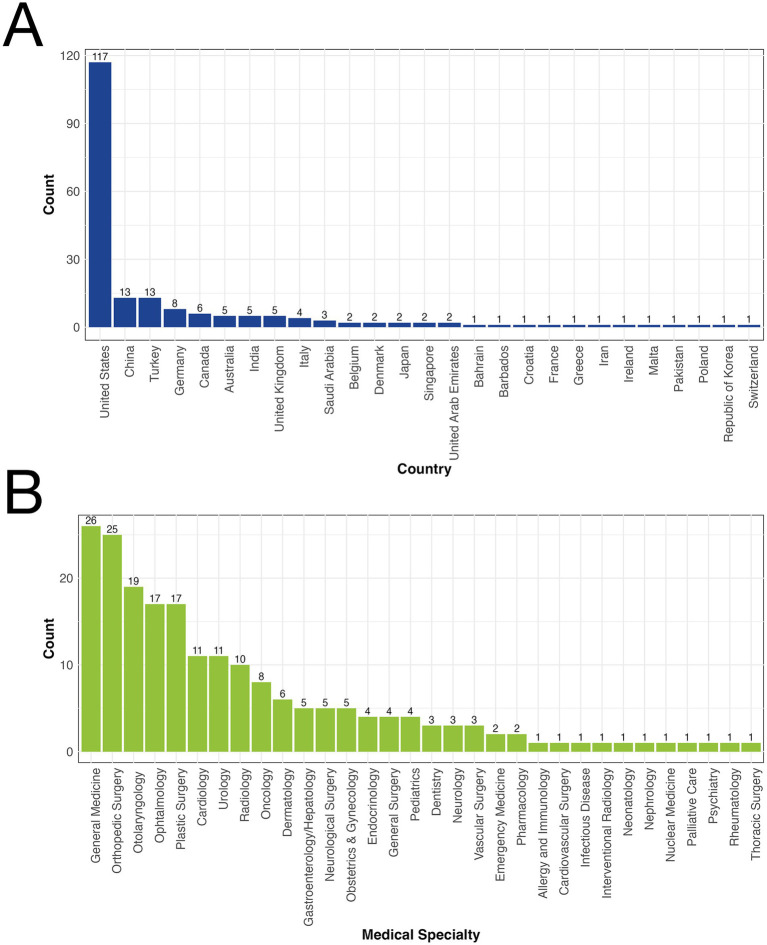
**(A)** Geographical distribution of studies on large language models (LLMs) in patient education. **(B)** Specialty distribution of studies on large language models (LLMs) in patient education.

The geographical distribution of the studies revealed a predominance from the United States, accounting for 58.2% (117/201) of the articles. Turkey and China followed, each contributing 6.4% (13/201) of the articles (Figure 1A). The studies spanned 35 medical specialties, with general medicine representing the largest proportion at 12.9% (26/201), closely followed by orthopedic surgery at 12.4% (25/201), and otolaryngology at 9.4% (19/201) (Figure 1B).

### 3.3 Thematic analysis

Our analysis identified six main themes with associated subthemes regarding the use of LLMs in patient education and engagement:

1. Generating Patient Education Materials
  - Answering Patient Questions
  - Enhancing Existing Patient Education Materials
  - Translation of Patient Education Materials
2. Interpreting Medical Information from a Patient Perspective
3. Providing Lifestyle Recommendations and Improving Health Literacy
4. Customized Medication Use and Self-Decision
5. Providing Pre-, Peri-, and Post-Operative Care Instructions
6. Optimizing Doctor-Patient Interaction
  - Facilitating Understanding of Consent Forms
  - Enhancing Communication Establishment

Table 1 presents these six themes as represented across the analyzed articles, along with illustrative quotes. Supplementary Data S2 indicates the theme to which each paper belongs.

**Table 1:** Representative quotes illustrating key themes identified in studies on the use of large language models (LLMs) in patient education.

Theme	Representative quotes
Generating Patient Education Materials Answering Patient Questions Enhancing Existing Patient Education Materials Translation of Patient Education Materials	**New Frontiers in Health Literacy: Using ChatGPT to Simplify Health Information for People in the Community** [Ayre et al. (159)] Ayre et al. evaluated ChatGPT-3.5’s ability to simplify health information for individuals with low literacy. The study found that ChatGPT effectively reduced text complexity by lowering the reading level, using simpler language, and decreasing passive voice usage. It retained about 80% of key messages, with more complex texts seeing greater improvements. However, most simplified texts still did not meet recommended health literacy targets. The researchers concluded that ChatGPT could provide a useful “first draft” of plain language health information, which could then be refined through human review. **Enhancing Readability of Online Patient-Facing Content: The Role of AI Chatbots in Improving Cancer Information Accessibility** [Abreu et al. (158)] Abreu et al. assessed ChatGPT-4’s effectiveness in improving the readability of cancer-related content from NCCN Member Institutions. The AI-generated outputs significantly reduced the reading level from university freshman to high school freshman level. This improvement in accessibility did not compromise content accuracy or quality. The simplified text featured shorter sentences and simpler words, earning a “good” quality rating on the DISCERN instrument. This study demonstrates AI’s potential to make complex medical information more accessible to patients. **Leveraging large language models for generating responses to patient messages** – **a subjective analysis** [Liu et al. (94)] Liu et al. compared fine-tuned LLaMA-based models (CLAIR-Short and CLAIR-Long) with ChatGPT in generating responses to patient messages. CLAIR-Long, fine-tuned with a mix of local patient messages and open-source data, performed comparably to ChatGPT-4 in empathy, responsiveness, and accuracy. CLAIR-Short, fine-tuned only with local data, produced concise responses similar to healthcare providers but less detailed. While ChatGPT-4 generally ranked highest, the study showed that fine-tuned models, especially CLAIR-Long, could be effective for patient education and empathetic communication. **Assessing the Accuracy and Reliability of AI-Generated Responses to Patient Questions Regarding Spine Surgery** [Kasthuri et al. (76)] Kasthuri et al. evaluated the GPT-4-enhanced Bing search engine’s responses to common spine surgery questions. Spine surgeons found the responses generally accurate and complete, with re-querying improving initially inaccurate answers. The study highlighted GPT-4-based models’ ability to provide useful summaries from web sources, but noted concerns about response quality variability. Most information came from commercial websites, with no significant correlation between response accuracy and source type. This research underscores the need for ongoing evaluation and refinement of LLMs for clinical use. **Easing the Burden on Caregivers-Applications of Artificial Intelligence for Physicians and Caregivers of Children with Cleft Lip and Palate** [Chaker et al. (199)] Chaker et al. tested ChatGPT-3.5’s ability to assist caregivers of children with cleft lip and palate. The AI achieved a 69% accuracy rate compared to senior pediatric plastic surgeons when answering common postoperative questions. While information-related errors were the AI’s main weakness, the study emphasized AI’s potential to ease caregiver burden by generating educational materials and offering perioperative support. This research highlights both the promise and current limitations of AI in specialized medical fields. **The utility of ChatGPT as a generative medical translator** [Grimm et al. (175)] Grimm et al. explored GPT-4’s utility in translating otolaryngology-related medical content into English, Spanish, and Mandarin. Using the Patient Education Materials Assessment Tool (PEMAT), they found that GPT-4 produced translations with comparable accuracy, understandability, and actionability across all three languages. This study suggests that LLMs like GPT-4 could play a valuable role in bridging language barriers in healthcare, potentially improving access to medical information for diverse patient populations.
Interpreting Medical Information from a Patient Perspective	**Quality of Answers of Generative Large Language Models Versus Peer Users for Interpreting Laboratory Test Results for Lay Patients: Evaluation Study** [He et al. (176)] He et al. conducted a comprehensive evaluation of several LLMs in interpreting laboratory test results for lay patients. The study compared GPT-4, GPT-3.5, LLaMA 2, MedAlpaca, and ORCA_mini across multiple metrics including accuracy, relevance, helpfulness, and safety. GPT-4 emerged as the top performer in all categories, followed closely by GPT-3.5. LLaMA 2, while providing detailed explanations, ranked third. MedAlpaca and ORCA_mini were less effective, with MedAlpaca showing the poorest performance. This study highlights the current superiority of GPT-4 and GPT-3.5 in translating complex medical information for patient understanding, suggesting their potential utility in healthcare communication. **Translating musculoskeletal radiology reports into patient-friendly summaries using ChatGPT-4** [Kuckelman et al. (82)] Kuckelman et al. explored GPT-4’s capability in simplifying musculoskeletal radiology reports for patients. The AI successfully generated summaries that were both readable and concise, with independent readers generally rating them as accurate and complete. GPT-4 demonstrated proficiency in simplifying medical jargon, making reports more accessible to patients. While there was some variation in accuracy and completeness ratings among readers, indicating a degree of subjectivity, the overall results were positive. The study suggests that GPT-4 could be a valuable tool in enhancing patient comprehension of radiology results, potentially reducing the immediate need for physician explanation. **Generative Artificial Intelligence to Transform Inpatient Discharge Summaries to Patient-Friendly Language and Format** [Zaretsky et al. (183)] Zaretsky et al. investigated GPT-4’s ability to transform complex inpatient discharge summaries into more patient-friendly formats. The AI-transformed summaries showed marked improvements in readability, with the Flesch–Kincaid Grade Level decreasing from 11.0 to 6.2. Understandability scores, measured by PEMAT, increased significantly from 13 to 81%. However, the study revealed mixed results in terms of accuracy and completeness. While 54% of reviews gave the highest accuracy rating, 18% identified safety concerns due to omissions or incorrect information (hallucinations). These findings indicate that while GPT-4 can greatly enhance the accessibility of discharge information, further refinement is necessary to ensure consistent accuracy and safety for practical use in healthcare settings.
Providing Lifestyle Recommendations and Improving Health Literacy	**Examining the role of ChatGPT in promoting health behaviors and lifestyle changes among cancer patients** [Alanezi et al. (184)] Alanezi et al. explored ChatGPT-3.5’s potential in promoting health behavior changes among cancer patients. The study found that the AI significantly improved health literacy, enhanced self-management practices, and provided valuable emotional and motivational support. Patients appreciated the AI’s ability to address their concerns, offer personalized suggestions, and connect them with relevant resources. However, the research also identified challenges, including privacy concerns, limitations in deep personalization, and occasional reliability issues. Despite these drawbacks, ChatGPT-3.5 proved effective in facilitating positive health behaviors and lifestyle changes, particularly in helping patients better understand and manage their conditions. **Assessing the Accuracy of Generative Conversational Artificial Intelligence in Debunking Sleep Health Myths: Mixed Methods Comparative Study With Expert Analysis** [Bragazzi et al. (185)] Bragazzi et al. assessed GPT-4’s accuracy in debunking common sleep-related myths. The AI correctly identified 85% of the presented myths as either “false” or “generally false,” demonstrating a sensitivity of 85% and a positive predictive value of 100%. GPT-4’s performance in identifying false statements was comparable to that of sleep experts, with high interrater agreement (ICC = 0.83). However, the AI sometimes struggled with nuanced scenarios, particularly myths containing partial truths or complex scientific concepts. The study concluded that while GPT-4 is a reliable tool for addressing sleep-related misinformation, it should not replace expert opinion in more nuanced areas. **Is ChatGPT an Effective Tool for Providing Dietary Advice?** [Ponzo et al. (190)] Ponzo et al. evaluated ChatGPT-3.5’s ability to provide accurate and appropriate dietary advice for various non-communicable diseases (NCDs). The AI’s advice was generally appropriate, with correctness rates ranging from 55.5% for sarcopenia to 73.3% for non-alcoholic fatty liver disease (NAFLD). However, the study revealed limitations in complex scenarios involving multiple overlapping conditions, where ChatGPT-3.5 sometimes provided contradictory or inappropriate recommendations. The researchers concluded that while ChatGPT-3.5 shows promise as a supplementary tool for dietary advice, it cannot yet replace personalized guidance from healthcare professionals, especially in managing complex cases.
Customized Medication Use and Self-Decision	**Snakebite Advice and Counseling From Artificial Intelligence: An Acute Venomous Snakebite Consultation With ChatGPT** [Altamimi et al. (192)] Altamimi et al. evaluated ChatGPT-3.5’s performance in providing information for managing venomous snakebites. The AI offered clear, evidence-based advice on initial first aid, the importance of seeking urgent medical attention, potential symptoms, and the role of antivenom. However, the study identified several limitations in the AI’s capabilities. These included a lack of personalization, outdated information, and an inability to account for regional variations in snake species and venom characteristics. While ChatGPT-3.5 proved effective in delivering general advice and preliminary guidance, the researchers emphasized that it should not replace professional medical consultations, especially in critical situations like snakebites. The study concluded by recommending future developments focus on addressing these limitations to enhance the AI’s utility in such scenarios. **Automating untruths: ChatGPT, self-managed medication abortion, and the threat of misinformation in a post-Roe world** [McMahon et al. (193)] McMahon et al. investigated the accuracy of ChatGPT-3.5’s responses regarding self-managed medication abortion (SMMA). The study revealed a concerning discrepancy in the AI’s information provision. While ChatGPT-3.5 correctly described clinician-managed medication abortion as safe and effective, it inaccurately portrayed SMMA as significantly more dangerous, exaggerating the risks of complications. This misrepresentation contradicts substantial evidence supporting SMMA’s safety and effectiveness. The researchers highlighted the potential dangers of such misinformation, noting it could increase stigma and deter individuals from seeking safe abortion methods, thereby posing a threat to public health. These findings emphasize the critical need for improving AI models to ensure they provide accurate and reliable health information, particularly on sensitive topics with significant public health implications.
Providing Pre-, Peri-, and Post-Operative Care Instructions	**Enhancing Postoperative Cochlear Implant Care With ChatGPT-4: A Study on Artificial Intelligence (AI)-Assisted Patient Education and Support** [Aliyeva et al. (194)] Aliyeva et al. evaluated ChatGPT-4’s effectiveness in providing postoperative care information for cochlear implant patients. The AI demonstrated high accuracy, clarity, and relevance in answering common postoperative questions. Its responses aligned well with current medical guidelines, ensuring patients received accurate and comprehensible information. The study found ChatGPT-4 to be a valuable supplementary resource, especially when access to healthcare professionals is limited. While emphasizing that ChatGPT-4 cannot replace professional medical advice, the researchers noted its potential to support patient education and reduce anxiety by providing timely information in resource-constrained settings. **Evaluation of large language model responses to Mohs surgery preoperative questions** [Breneman et al. (206)] Breneman et al. compared the performance of three large language models (ChatGPT-3.5, Google Bard, and Microsoft CoPilot) in answering preoperative questions about Mohs surgery. ChatGPT-3.5 outperformed the other models in accuracy (80%) and completeness (100%) of responses. However, its higher reading level (12.7) potentially made the information less accessible to some patients. Google Bard and Microsoft CoPilot, while less accurate and complete, provided more readable responses. The study highlighted the potential of LLMs like ChatGPT-3.5 in offering valuable preoperative information but cautioned about possible inaccuracies or irrelevant details, emphasizing the need for careful implementation in patient education. **Feasibility of GPT-3 and GPT-4 for in-Depth Patient Education Prior to Interventional Radiological Procedures: A Comparative Analysis** [Scheschenja et al. (195)] Scheschenja et al. conducted a comparative analysis of GPT-3 and GPT-4 in providing patient education for interventional radiology procedures. GPT-4 showed superior performance, with 35.3% of its responses rated as “completely correct” compared to GPT-3’s 30.8%. GPT-4 also had fewer “mostly incorrect” responses (2.3% vs. GPT-3’s 5.3%). Despite these differences, both models were considered safe and effective for patient education, with GPT-4 having a slight edge. The researchers concluded that while these AI tools can enhance patient understanding of complex procedures, they should be used cautiously due to the potential for inaccuracies or incomplete information.
Optimizing Doctor-Patient Interaction Facilitating Understanding of Consent Forms Enhancing Communication Establishment	**Bridging the literacy gap for surgical consents: an AI-human expert collaborative approach** [Ali et al. (208)] Ali et al. investigated the use of GPT-4 to simplify surgical consent forms, aiming to make them more accessible to patients with varying health literacy levels. The study found that GPT-4 significantly improved the readability of consent forms from 15 academic medical centers, reducing the average reading level from college freshman to 8th-grade level. Moreover, GPT-4 generated procedure-specific consent forms that maintained medical and legal sufficiency, scoring perfectly on a validated rubric and passing expert review without changes. This research demonstrates the potential of AI-human collaboration in enhancing the clarity and comprehensibility of consent forms, ensuring patients receive clear, detailed information about their surgical procedures. **Generating Informed Consent Documents Related to Blepharoplasty Using ChatGPT** [Shiraishi et al. (209)] Shiraishi et al. evaluated ChatGPT’s performance in generating informed consent (IC) documents for blepharoplasty. While the study showed promise for LLMs in enhancing patient communication, it also highlighted areas needing improvement. Board-certified plastic surgeons rated AI-generated documents lower than original IC documents in accuracy, informativeness, and accessibility. Even after revisions, the AI-generated documents still scored lower in accuracy and accessibility. Interestingly, nonmedical staff found no significant difference between AI-generated and original documents. The study concluded that while ChatGPT has potential, it currently cannot replace human-generated IC documents due to issues with professional terminology and content accuracy, emphasizing the need for further refinement. **Putting ChatGPT’s Medical Advice to the (Turing) Test: Survey Study** [Nov et al. (110)] Nov et al. assessed laypeople’s ability to distinguish between medical advice from ChatGPT-3.5 and human healthcare providers. Participants could only weakly differentiate between the sources, correctly identifying them about 65% of the time. Trust in ChatGPT-3.5’s responses decreased with increasing medical complexity of the questions, with higher trust in logistical responses and lower trust in diagnostic and treatment-related responses. The study concluded that while ChatGPT-3.5 can provide credible advice for low-risk queries, it may not be reliable for more complex health issues, suggesting the need for further research to optimize its use in patient-provider communications. **Can Large Language Models Generate Outpatient Clinic Letters at First Consultation That Incorporate Complication Profiles From UK and USA Aesthetic Plastic Surgery Associations?** [Roberts et al. (211)] Roberts et al. compared ChatGPT-4, ChatGPT-3.5, and Google Bard in generating outpatient clinic letters incorporating complication profiles from aesthetic plastic surgery associations. ChatGPT-4 showed the highest overall compliance, scoring 0.92 for BAAPS and 0.99 for ASPS compliance. However, its performance dropped to 0.52 for ASPS gold-standard profiles, indicating challenges with paywalled content. ChatGPT-3.5 and Google Bard demonstrated lower compliance overall. This study highlights the potential of advanced LLMs in generating compliant medical documentation, while also revealing limitations in accessing and integrating specialized, restricted information.

The theme “Generating Patient Education Materials” was predominant, encompassing 80.5% (162/201) of the articles across its three subthemes. Within this theme, “Answering Patient Questions” was the most prevalent subtheme, representing 71.6% (144/201) of all articles. The remaining themes were distributed as follows: “Interpreting Medical Information from a Patient Perspective” and “Providing Lifestyle Recommendations and Improving Health Literacy” each accounted for 4.5% (9/201) of the articles. “Providing Pre-, Peri-, and Post-Operative Care Instructions” was represented in 6.9% (14/201) of the articles, while “Optimizing Doctor-Patient Interaction” appeared in 2.5% (5/201) of the articles. The least represented theme was “Customized Medication Use and Self-Decision,” accounting for 1% (2/201) of the articles.

#### 3.3.1 Theme 1: generating patient education materials

The generation of patient education materials emerged as a prominent theme, with three key subthemes: answering patient questions, enhancing existing materials, and translating medical content. Answering patient questions was the most significant subtheme, representing 71.6% of the articles (8, 15–157). In these studies, LLMs created educational content by responding to common questions, direct patient inquiries, and expert-formulated queries, demonstrating their potential to address diverse patient information needs.

Most studies found LLMs provided accurate responses to patient queries. Almagazzachi et al. reported 92.5% accuracy for ChatGPT’s answers to hypertension questions (18). However, accuracy varied by specialty. In a study on pediatric in-toeing, Amaral et al. found 46% of responses were excellent, and 44% were satisfactory with minimal clarification needed (19). These findings suggest LLMs’ potential in patient education, while highlighting performance differences across medical fields.

The readability of LLM-generated content varied considerably across studies. ChatGPT’s responses often required a higher reading level, potentially limiting accessibility for some patients. Campbell et al. demonstrated that ChatGPT’s unprompted answers on obstructive sleep apnea had a mean Flesch–Kincaid grade level of 14.15, which decreased to 12.45 when prompted (32). This indicates that even with specific instructions, the content remained at a college reading level. In contrast, other LLMs showed better readability in some cases. Chervonski et al. reported that Google BARD produced more accessible content, with responses on vascular surgery diseases achieving a mean Flesch Reading Ease score of 58.9, indicating improved readability (40). When compared to traditional search engines, LLMs revealed a trade-off between comprehensiveness and readability. Cohen et al. found that while ChatGPT provided more detailed and higher-quality responses to cataract surgery FAQs compared to Google, these responses were at a higher reading level (42). These findings suggest that while LLMs may offer more comprehensive information, they do not always improve accessibility for the average patient.

LLMs show promise in transforming existing materials into more readable, patient-centered formats (158–174). Numerous studies demonstrate their ability to enhance readability across various medical education materials (158–161, 163–165, 168, 170–172, 174). Fanning et al. found comparable performance between ChatGPT-3.5 and ChatGPT-4 in improving plastic surgery material readability (166). Moons et al. reported Google BARD surpassed GPT in readability improvement but tended to omit information (169). Some studies, however, found no improvement or decreased readability (162, 167), indicating variability in LLM effectiveness. Interestingly, Sudharshan et al. noted LLMs were more accurate in creating readable Spanish materials (173), suggesting potential for addressing language-specific challenges.

Research on LLMs for translating patient education materials remains limited. However, a significant study by Grimm et al. showed ChatGPT-4’s ability to produce accurate, understandable, and actionable translations of otorhinolaryngology content in English, Spanish, and Mandarin (175). This finding suggests LLMs’ potential in overcoming language barriers in patient education.

#### 3.3.2 Theme 2: interpreting medical information from a patient perspective

Nine studies investigated LLMs’ capacity to interpret complex medical information, evaluating their feasibility, accuracy, readability, and effectiveness in translating medical jargon. He et al. found ChatGPT-4 outperformed other LLMs and human responses from Q&A websites in accuracy, helpfulness, relevance, and safety when answering laboratory test result questions (176). However, Meyer et al. reported that ChatGPT, Gemini, and Le Chat were less accurate and more generalized than certified physicians in interpreting laboratory results (177), highlighting the variability in LLM performance across different contexts.

LLMs demonstrate potential in improving radiological information interpretation and communication. Kuckelman et al. found ChatGPT-4 produced generally accurate summaries of musculoskeletal radiology reports, noting some variability in human interpretation (82). Lyu et al. showed ChatGPT-4 enhanced translated radiology report quality and accessibility, despite occasional oversimplifications (178). Sarangi et al. reported ChatGPT-3.5 effectively simplified radiological reports while maintaining essential diagnostic information, though performance varied across conditions and imaging modalities (179). Several other studies support these findings, suggesting LLMs’ promising role in radiology communication (180–182).

Zaretsky et al. evaluated ChatGPT-4’s ability to convert discharge summaries into patient-friendly formats. The transformed summaries showed significant improvements in readability and understandability. However, the study raised concerns about accuracy and completeness, noting instances of omissions and hallucinations (183).

#### 3.3.3 Theme 3: providing lifestyle recommendations and improving health literacy

Nine studies explored LLMs’ potential in offering lifestyle recommendations and enhancing health literacy. Alanezi et al. found ChatGPT effective in promoting health behavior changes among cancer patients, boosting health literacy and self-management (184). Bragazzi et al. showed ChatGPT’s capability to debunk sleep-related myths and provide accessible advice (185). In a follow-up study, they found Google BARD slightly outperformed ChatGPT-4 in identifying false statements and offering practical sleep-related advice (186). These findings suggest LLMs’ promising role in health education and lifestyle guidance.

Gray et al. demonstrated ChatGPT’s ability to generate realistic prenatal counseling dialogues (187). Minutolo et al. proposed a conversational agent to enhance health literacy by making Patient Information Leaflets queryable (188). Mondal et al. found ChatGPT provided reasonably accurate responses to lifestyle-related disease queries (189). Ponzo et al. reported ChatGPT offered general dietary guidance for NCDs but struggled with complex, multi-condition cases (190). Willms et al. explored ChatGPT’s potential in creating physical activity app content, emphasizing the need for expert review (1). Zaleski et al. found AI-generated exercise recommendations generally accurate but lacking comprehensiveness and at a college reading level (191). These studies highlight LLMs’ diverse applications in health education while noting their limitations.

#### 3.3.4 Theme 4: customized medication use and self-decision

Two studies explored LLMs’ potential in medication guidance and self-decision support. Altamimi et al. found ChatGPT provided accurate advice on acute venomous snakebite management, while emphasizing the importance of professional care (192). In contrast, McMahon et al. observed ChatGPT accurately described clinician-managed abortion as safe but incorrectly portrayed self-managed abortion as dangerous, highlighting potential misinformation risks (193). These findings underscore both the promise and pitfalls of using LLMs for sensitive medical information.

#### 3.3.5 Theme 5: providing pre-/peri-/post-operative care instructions

Studies investigated LLMs’ use in surgical patient education. Aliyeva et al. found ChatGPT-4 excelled in providing postoperative care instructions for cochlear implant patients, especially in remote settings (194). LLMs showed proficiency in offering postoperative guidance across various surgical specialties (180, 195–202). Dhar et al. noted ChatGPT’s accuracy in answering tonsillectomy questions, with some pain management inaccuracies (203). Patil et al. reported ChatGPT provided quality preoperative information for ophthalmic surgeries, though occasionally overlooking adverse events (204). Meyer et al. found ChatGPT reliable for postoperative gynecological surgery instructions (205). Breneman et al. and Kienzle et al. evaluated ChatGPT for preoperative counseling in Mohs surgery and knee arthroplasty, finding it potentially useful but cautioning about non-existing references (206, 207).

#### 3.3.6 Theme 6: optimizing doctor-patient interaction

This theme explores LLMs’ potential to enhance doctor-patient communication, particularly in simplifying consent forms and improving general medical communication. Ali et al. found ChatGPT-4 successfully simplified surgical consent forms to an 8th-grade reading level while maintaining accuracy (208). Shiraishi et al. reported that revised ChatGPT-prepared informed consent documents for blepharoplasty were more desirable than originals (209).

LLMs also showed promise in broader doctor-patient communication. An et al. introduced an LLM-based education model that improved patients’ understanding of their conditions and treatments (210). Roberts et al. demonstrated LLMs could generate comprehensible outpatient clinic letters for cosmetic surgery, potentially saving clinicians’ time (211). Xue et al. found ChatGPT performed well in logical reasoning and medical knowledge education during remote orthopedic consultations (212). These studies highlight LLMs’ potential to enhance various aspects of medical communication.

## 4 Discussion

This scoping review synthesizes current applications and potential uses of LLMs in patient education and engagement, offering insights into their transformative potential and integration challenges in healthcare settings. LLMs demonstrate significant promise in creating patient education materials, with studies reporting that health-related questions were accurately answered over 90% of the time by systems like ChatGPT, covering a broad range of topics from hypertension to pediatric conditions (18, 31). The depth of these responses potentially offers substantial value to patients seeking detailed understanding of their ailments. However, readability remains a notable concern, potentially limiting accessibility for some patient populations.

LLMs have demonstrated competence in interpreting complex medical information from laboratory reports, radiology results, and discharge summaries. ChatGPT-4, for instance, generated informative summaries of radiology reports, making them more accessible to non-medical professionals (82, 178). However, concerns about the quality and comprehensiveness of LLM-generated information persist. Issues such as hallucinations, omissions, or plausible but incorrect information have been noted. Zaretsky et al. observed that while ChatGPT-4 could transform discharge summaries into more patient-friendly formats, occasional inaccuracies, and omissions could potentially mislead patients (183). These findings underscore the necessity for professional oversight in deploying LLMs in healthcare settings to ensure the reliability and accuracy of AI-generated content.

LLMs show promise as lifestyle recommendations and health literacy tools, effectively encouraging healthy behaviors and dispelling health myths. Alanezi et al. found that ChatGPT provided significant support in developing health literacy among cancer patients, motivating self-management through emotional, informational, and motivational assistance (184). Bragazzi and Garbarino demonstrated ChatGPT’s effectiveness in debunking sleep-related misconceptions, accurately distinguishing between false and genuine health information (185). However, personalization and accuracy remain challenging. While AI can offer useful preliminary advice, it requires further development to provide relevant, situation-specific suggestions tailored to individual patients. This customization is crucial for ensuring that patients can trust and adhere to the recommendations provided.

LLMs play a significant role in providing information on self-medication and personalized drug utilization, offering detailed insights on drug interactions, correct usage, and potential side effects. Altamimi et al. found ChatGPT’s information helpful and accurate in guiding acute venomous snakebite management, though it appropriately emphasized the need for professional medical care (192). LLMs also show potential in patient triage, quickly analyzing symptoms and medical history to prioritize cases based on severity (10). However, the quality of LLM-provided information varies considerably. McMahon et al. reported that ChatGPT gave inaccurate and misleading information about self-managed medication abortion, incorrectly portraying it as dangerous despite evidence of its safety and efficacy (193). This inconsistency highlights the risks of relying on AI without professional oversight and underscores the need for LLMs to provide accurate, up-to-date, and context-sensitive information to support safe self-medication practices.

### 4.1 Implications and future research

The integration of LLMs into patient education and engagement shows significant potential for improving health literacy and healthcare delivery efficiency. However, this review highlights the need for continued improvement in the accuracy and personalization of AI-generated content. Future research should focus on developing more accurate LLM algorithms to enhance reliability as medical information sources, exploring multimodal LLMs, and establishing robust validation frameworks for their ethical use. Ensuring AI-based information aligns with the latest medical guidelines and is tailored for diverse patient populations is crucial. Conducting longitudinal studies to assess the long-term effects of LLMs on patient outcomes and satisfaction will provide valuable insights. Additionally, addressing ethical concerns, including data privacy and potential biases in LLM-generated content, is essential. These research directions are crucial for the responsible and effective integration of LLMs in healthcare settings. Finally, LLMs may carry biases from their training data, potentially propagating misinformation or reinforcing healthcare disparities. Future research should address these limitations by ensuring LLM tools are accurate, reliable, and equitable across diverse patient populations, while also exploring their long-term effects and ethical implications.

### 4.2 Limitations

This scoping review has several limitations. The quality of included studies varied, with some using small sample sizes or subjective assessments, potentially limiting result generalizability. Most studies were conducted in high-income countries, raising questions about their relevance to low-and middle-income settings with different healthcare needs and infrastructure. The evaluation of various LLMs and versions complicates drawing overarching conclusions. Inconsistent evaluation metrics across studies hindered result comparison and synthesis.

## 5 Conclusion

LLMs demonstrate transformative potential in patient education and engagement across various levels of medical care. Their ability to provide accurate, detailed, and timely information can significantly enhance patients’ understanding of their healthcare and promote active involvement. However, current limitations in accuracy and readability highlight the need for further refinement to ensure reliable integration with healthcare systems. Extensive research and development of AI tools are necessary to fully harness their potential for improving patient outcomes and healthcare efficiency. A critical priority for medical applications is to ensure the ethical and responsible use of these tools, necessitating robust supervision and validation processes.
