# Supplementary material for: Large language models in patient education: a scoping review of applications in medicine
Source: Front Med (Lausanne). 2024 Oct 29;11:1477898. doi: 10.3389/fmed.2024.1477898 (PMC11554522; doi:10.3389/fmed.2024.1477898)
Supplement: Supplementary file 3 [file Image_1.pdf]

Supplementary Material

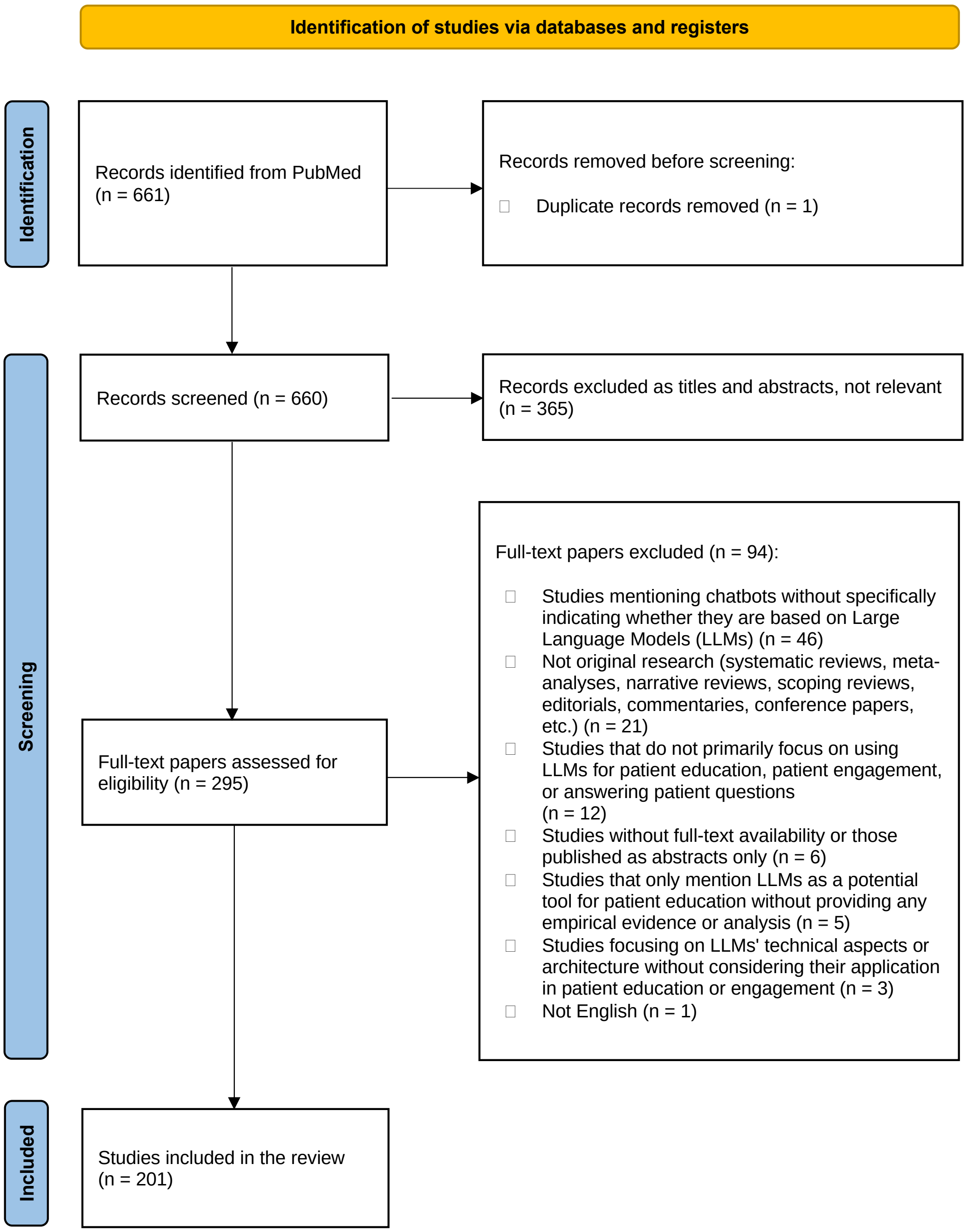

From: Page MJ, McKenzie JE, Bossuyt PM, Boutron I, Hoffmann TC, Mulrow CD, et al. The PRISMA 2020 statement: an updated guideline for reporting systematic reviews. BMJ 2021;372:n71. doi: 10.1136/bmj.n71

Supplementary Figure 1. PRISMA Flow Diagram.
