# Supplementary material for: Large language models in patient education: a scoping review of applications in medicine
Source: Front Med (Lausanne). 2024 Oct 29;11:1477898. doi: 10.3389/fmed.2024.1477898 (PMC11554522; doi:10.3389/fmed.2024.1477898)
Supplement: Supplementary file 4 [file Table_1.docx]

Supplementary Material

**Supplementary Methods 1.** Full search strategy for PubMed.

| **Set #** | **PubMed** |
| --- | --- |
| **#1 Patient Education/Engagement** | *("Patient Education as Topic"[MeSH] OR "patient educ*"[tiab] OR "patient train*"[tiab] OR "patient involv*"[tiab] OR "patient quest*"[tiab] OR "health education*" OR "health literacy"[tiab] OR "patient learn*"[tiab] OR "patient aware*"[tiab] OR "patient instruction*"[tiab] OR "patient communicat*"[tiab] OR "patient support*"[tiab])* |
| **#2 LLM** | *("Artificial Intelligence"[MeSH] "artificial intel*"[tiab] OR "language model*"[tiab] OR llm[tiab] OR "generative ai*"[tiab] OR "generative artificial intel*"[tiab] OR gpt[tiab] OR chatgpt*[tiab] OR bard*[tiab] OR claude*[tiab] OR perplexity*[tiab] OR llama*[tiab] OR chatbot*[tiab] OR "conversational ai*"[tiab] OR "conversational artificial intel*"[tiab])* |
| **#3** | #1 AND #2 |
