# Supplementary material for: Large language models in patient education: a scoping review of applications in medicine
Source: Front Med (Lausanne). 2024 Oct 29;11:1477898. doi: 10.3389/fmed.2024.1477898 (PMC11554522; doi:10.3389/fmed.2024.1477898)
Supplement: Supplementary file 5 [file Table_2.docx]

Supplementary Material

**Supplementary Material 2.** Detailed eligibility criteria.

***Inclusion Criteria***

1. Studies that investigate the use, accuracy, relevance, or effectiveness of large language models (LLMs) (e.g., ChatGPT, Google Bard, Claude) specifically for patient education, patient engagement, answering patient-specific questions, or generating patient education materials.
2. Studies where the LLM output is aimed at patients, with content evaluated from the patient’s perspective.
3. Studies involving direct interactions between patients and LLMs or scenarios where LLM-generated content is intended for patient use.
4. Studies that evaluate specific outcomes related to patient education and training, such as improvements in patient knowledge, adherence to treatment, patient satisfaction, and health literacy.
5. Peer-reviewed original research, including qualitative, quantitative, and mixed-methods studies.
6. Studies published in English.
7. Studies with full-text availability.

***Exclusion Criteria***

1. Studies that do not primarily focus on the use of LLMs for patient education, patient engagement, or answering patient questions.
2. Studies evaluating LLMs in contexts other than healthcare or patient-related applications.
3. Studies mentioning chatbots without specifically indicating whether they are based on Large Language Models (LLMs)
4. Studies focus on the technical aspects or architecture of LLMs without considering their application in patient education or engagement.
5. Studies that only mention LLMs as a potential tool for patient education without providing empirical evidence or analysis.
6. Systematic reviews, meta-analyses, narrative reviews, scoping reviews, editorials, commentaries, conference papers, and other non-original research articles.
7. Studies published in languages other than English.
8. Studies without full-text availability or those published as abstracts only.
